# Uncovering the diversity of endemic Ethiopian fauna: complete mitochondrial genomes of four *Lophuromys* species (Rodentia, Muridae)

**DOI:** 10.1080/23802359.2022.2079435

**Published:** 2022-06-14

**Authors:** Valeria A. Komarova, Nikolai S. Mugue, Danila S. Kostin, Leonid A. Lavrenchenko

**Affiliations:** a Laboratory of Mammalian Microevolution, A.N. Severtsov Institute of Ecology and Evolution RAS, Moscow, Russia; b Laboratory of Molecular Genetics, Russian Federal Research Institute of Fisheries and Oceanography, Moscow, Russia

**Keywords:** *Lophuromys*, Ethiopia, mitochondrial DNA, Muridae phylogeny

## Abstract

Complete mitochondrial genomes of four species of Ethiopian speckled brush-furred rats *Lophuromys* (*L. chrysopus*, *L. menageshae, L. melanonyx*, and *L. simensis*) were assembled for the first time. We provide data concerning the sequencing, assembly, and annotation of the obtained mitogenomes; compare two widely used circular-genome annotation tools (MITOS and MitoZ), and discuss relevant points concerning relationships within both Ethiopian *Lophuromys* and the Muridae family.

## Introduction

Ethiopian speckled brush-furred rats of the genus *Lophuromys* belong to the *L. flavopunctatus* species complex, which is widespread in Ethiopia and consists of nine species that are well-delimited both morphologically and genetically (Lavrenchenko et al. 2007; Bryja et al. 2019). The complex has emerged and flourished in the Ethiopian highlands, which are characterized by a patchwork of climatic conditions (Bryja et al. 2018). These circumstances have resulted in a process of adaptive radiation through so-called ‘reticulate’ evolution, which features multiple exchanges of genomic segments, primarily mitochondrial genomes, between species (Lavrenchenko et al. 2004; Komarova et al. 2021). As a consequence, among Ethiopian representatives of the genus, one can more or less clearly distinguish traces of at least four such introgression events, and some of them represent a complete replacement of the mitochondrial genome (Kostin et al. 2019). Despite an ever-increasing number of complete mitochondrial genomes, publicly available in the GenBank database, until the current study there was no mitochondrial genome data for none of *Lophuromys* genus representatives but only single *cytb* sequences (1140 bp). To fill this gap, we present here complete mitochondrial genomes of four out of the nine Ethiopian Lophuromys species [L. chrysopus Osgood, 1936; L. menageshae Lavrenchenko et al., 2007; L. melanonyx Petter, 1972 and L. simensis Osgood, 1936]. It is worth noting that in populations of the last two species, aside from the apparently species-specific mitotypes presented here, there is another one, presumably derived from *L. menageshae* [Kostin et al. (2019); for more details, kindly refer to Komarova et al. (2021)].

## Materials and methods

**Figure 1.**
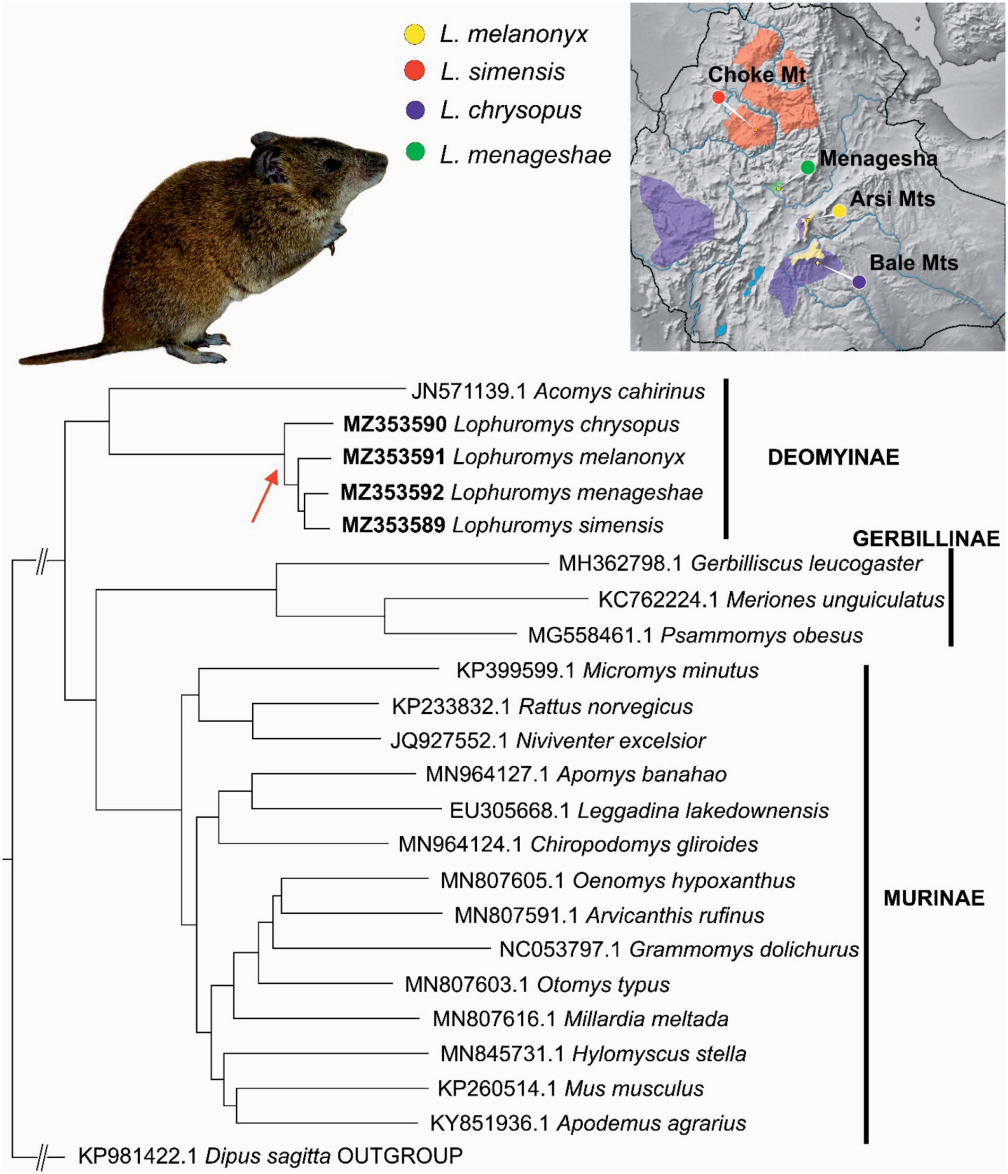
The maximum-likelihood phylogenetic tree constructed in IQTREE with 1000 bootstrap replications. Bootstrap supports of all nodes are >90. Red arrow shows the position of the Ethiopian *Lophuromys* on the phylogenetic tree. Colored map: schematic representation of geographic ranges of the four *Lophuromys* species. The circles show the voucher specimens’ collection sites.

Muscle tissues were collected during the Joint Ethio-Russian Biological Expedition in 1998 (*L. menageshae*, Ethiopia, Menagesha forest, 8.95 N, 38.55 E), 2013 (*L. chrysopus,* Ethiopia, Bale Mts., Harenna forest, 6.645 N, 39.733 E), 2015 (*L. melanonyx*, Ethiopia, Arsi Mts., 7.825 N, 39.412 E,) and 2018 (*L. simensis*, Ethiopia, Choqe Mt., 10.7058 N, 37.8432 E) (see also Figure 1, colored map). All experimental procedures were carried out in accordance with relevant guidelines in compliance with the International Union for Conservation of Nature (IUCN) policies regarding research involving species at risk of extinction (for details see Guidelines for appropriate uses of IUCN Red list data). All voucher specimens were deposited in the collection of the Zoological Museum of Lomonosov Moscow State University (https://zmmu.msu.ru, Vladimir S. Lebedev, wslebedev@mail.ru) under ID numbers S-165969 (*L. menageshae*), S-192730 (*L. chrysopus*), S-197618 (*L. melanonyx*) and S-202836 (*L. simensis*).

DNA was extracted using a commercial kit (Jena Bioscience) according to the manufacturer’s instructions. Total genomic DNA was sonicated, and after adapter ligation, was sequenced on the Illumina HiSeq platform. Complete mitochondrial genomes were assembled by means of NOVOplasty 4.3.1 (Dierckxsens et al. 2017) with available sequences of the *cytb* gene serving as a starting seed. Because one of the aims of this study was the comparison of available annotation software, we tested two popular tools: MITOS (Bernt et al. 2013) and MitoZ (Meng et al. 2019). Finally, using complete mitochondrial sequences (available in GenBank) from 18 representatives of the Muridae family and the sequence of the northern three-toed jerboa (*Dipus sagitta*, Pallas, 1773) as an outgroup, we performed phylogenetic reconstruction by the maximum-likelihood approach in IQTREE (Nguyen et al. 2015) using 1000 bootstrap replications to assess robustness of the nodes.

## Results and discussion

The complete mitogenomes of all four species were found to have a similar composition (*L. simensis* – 16,277 bp, *L. chrysopus* – 16,276 bp, *L. melanonyx* – 16,273 bp, and *L. menageshae* – 16,274 bp.) and contain 22 transfer RNA (tRNA) genes, two ribosomal-RNA genes (12S rRNA and 16S rRNA), 13 protein-coding genes (PCGs) and one non-coding control region. The overall nucleotide composition of the *L. menageshae* genome (in the other three species, it is virtually identical, with slight differences) is 33.7% of A, 26.6% of T, 12.2% of G, and 27.5% of C. The total length of the 13 PCGs is 11,310 bp. Initiation codons for all 13 PCGs can be described as ATN: ATA for *nad1*, *nad2*, and *nad5*; ATG for *cox1*, *cox2*, *atp8*, *atp6*, *cox3*, *nad3* (*L. chrysopus*), *nad4l*, *nad4*, *nad6*, and *cytb*; and ATC for *nad2* (*L. simensis*) and *nad3* (*L. chrysopus*). Termination codons for all 13 PCGs are represented by two typical variants: either TAA or TAG. Both annotation tools (MITOS and MitoZ) revealed that the obtained mitogenomes are identical in structure (2 rRNA genes, 13 PCGs, and 22 tRNAs genes). Nonetheless, we found that MITOS has a weaker capacity for precise determination of gene length, especially at an end. For example, all 13 PCG sequences annotated by MITOS were shorter by 1–4 amino acid residues at the 3′ end. It is also worth mentioning that this MITOS drawback does not allow to deposit of obtained sequences in the GenBank database owing to the erroneous annotation. Until we identify the cause of errors in termination codon identification in MITOS, we cannot recommend this tool for annotation purposes and instead give our preference to MitoZ as more accurate. The mitogenomes annotated by means of MitoZ as well as the raw sequence reads were deposited in GenBank (see accession numbers below).

The resultant phylogenetic reconstruction (Figure 1) revealed the following pattern of relationships among the species: the basal position is occupied by *L. chrysopus*, and the next split divided *L. melanonyx* from the sister pair *L. menageshae* and *L. simensis*. In a comparison with a recently published Ethiopian *Lophuromys* study (Komarova et al. 2021) – based on a set of ddRadSeq SNPs and sequences of the *cytb* gene – our data turned out to be more similar to the topology of the ddRadSeq SNP tree (‘*chrysopus*’(‘*melanonyx*’(‘*menageshae*’ & ‘*simensis*’))) than to the tree based on a single mitochondrial gene (*cytb*, 1140 bp): (‘*chrysopus*’(‘*simensis*’(‘*melanonyx*’ & ‘*menageshae*’))). Taking into account quite recent diversification of Ethiopian *Lophuromys* [no later than ca. 1.4 million years ago; see Komarova et al. (2021)], one can assume that the phylogenetic signal in the stand-alone mitochondrial gene (*cytb*) is not sufficient to resolve phylogenetic relationships between closely related species that have emerged from through rapid adaptive radiation. This observation points to the applicability of complete mitogenome sequences to the unraveling of shallow phylogenetic relationships below the genus level.

The other point of interest is that the obtained topology of the Muridae family is different from the generally accepted one [for example, see studies by Alhajeri et al. (2015) and Aghová et al. (2018) conducted on the basis of multilocus data including both nuclear and mitochondrial gene fragments]. Instead of expected sister relationships of subfamilies Deomyinae and Gerbillinae, our data are suggestive of a basal position of Deomyinae and proximity of Gerbillinae to Murinae (Figure 1). It should be noted that the latter pattern is in agreement with a recently published paper (Song et al. 2021), where on the basis of mitochondrial sequences of 13 PCGs and two rRNAs, a similar topology was shown. Considering that the reconstruction of such deep diversification events by means of complete mitochondrial sequences may yield results that are erroneous or characterized by low bootstrap support, we have no choice but wait for the publication of nuclear genomic data, which are expected to resolve this kind of issues [for a similar example, see Mikula et al. (2021)].

Despite the limited utility of the small number of mitogenome sequences, the gradual accumulation of a substantial set of sequences from non-model species opens up big opportunities for further research. This is especially true in the case of such groups as *Lophuromys*, where previously described putatively adaptive introgression events (Komarova et al. 2021) require more detailed and data-intensive investigation. Last but not least, it is important to note the narrowness of geographic ranges of Ethiopian *Lophuromys* species, especially for the obligate forest dweller *L. menageshae* (Figure 1, colored map). In view of the accelerating pace of anthropogenic transformation, it is easy to predict that *Lophuromys* populations or even whole species [such as *L. melanonyx*, currently listed as vulnerable by the International Union for Conservation of Nature (Kennerley and Lavrenchenko 2016)] will face the threat of extinction.

## Data Availability

The genome sequence data that support the findings of this study are openly available in GenBank of the NCBI (https://www.ncbi.nlm.nih.gov/) under the accession numbers MZ353589 – MZ353592. The associated BioProject, SRA and Bio-Sample numbers are PRJNA763532; SRR15904659, SRR15904661, SRR15904660 and SRR15904662; and SAMN21442456, SAMN21442454, SAMN21442455 and SAMN21442453, respectively.
